# ADAMTS4 is a serum biomarker for pulmonary arterial hypertension associated with congenital heart disease

**DOI:** 10.3389/fmed.2026.1863052

**Published:** 2026-07-22

**Authors:** Genfa Xiao, Yuanrong Chen, Ying Meng, Ziliang Wang, Jiumei Cai, Zuofeng Cao, Shixiu Zeng, Weihua Liang, Yueling Yu, Haobo Wang, Lijun Nie, Xiaoli Liu, Yongling Liao

**Affiliations:** 1 Department of Cardiology, Heart Medical Centre, First Affiliated Hospital of Gannan Medical University, Ganzhou, China; 2 Key Laboratory of Prevention and Treatment of Cardiovascular and Cerebrovascular Diseases, Ministry of Education, First Affiliated Hospital of Gannan Medical University, Ganzhou, China; 3 First Clinical Medical College of Gannan Medical University, Ganzhou, China

**Keywords:** ADAMTS4, biomarker, diagnosis, prognosis, pulmonary arterial hypertension associated with congenital heart disease

## Abstract

**Objectives:**

Pulmonary arterial hypertension associated with congenital heart disease (PAH-CHD) is a cardiopulmonary disorder characterized by pathological pulmonary vascular remodeling and perivascular inflammatory cell infiltration. ADAMTS4 is a secreted, zinc-dependent metalloprotease critically involved in extracellular matrix degradation and turnover and has recently emerged as a potential biomarker in vascular remodeling diseases. This study aimed to evaluate the diagnostic performance and prognostic value of serum ADAMTS4 in patients with PAH-CHD.

**Methods:**

This study included patients with PAH-CHD, patients with CHD, and healthy controls. Serum ADAMTS4 levels were measured by ELISA. Correlations with clinical parameters were assessed using Spearman’s rank correlation. The diagnostic efficacy was evaluated by ROC curve analysis. Univariate and multivariable logistic regression were used to identify independent risk factors associated with the presence of PAH-CHD. PAH-CHD patients were followed up for a median duration of 40.5 months, and Kaplan–Meier and Cox regression analyses were used to analyze prognosis.

**Results:**

Compared with those in CHD patients and healthy controls, serum ADAMTS4 levels were elevated in PAH-CHD patients. ADAMTS4 was positively correlated with NT-proBNP (*r* = 0.45, *p* = 0.002), CRP (*r* = 0.60, *p* < 0.001), and WHO functional class (*r* = 0.49, *p* < 0.001). ROC analysis demonstrated good discriminatory ability for differentiating PAH-CHD from CHD (AUC = 0.78; 95% CI: 0.69–0.87; *p* < 0.001), with an optimal cutoff value of 42.2 ng/mL. After multivariable adjustment, logistic regression revealed that ADAMTS4 remained an independent factor associated with the presence of PAH-CHD (OR = 1.189; 95% CI: 1.036–1.365; *p* = 0.014). Kaplan–Meier analysis revealed significantly shorter event-free survival in patients with ≥ 42.2 ng/mL ADAMTS4 than in those with lower levels (log-rank *p* = 0.046). After multivariable adjustment, Cox regression confirmed ADAMTS4 as an independent predictor of adverse clinical events (HR = 1.038; 95% CI: 1.007–1.071; *p* = 0.017).

**Conclusion:**

Serum ADAMTS4 is elevated in patients with PAH-CHD and is correlated with disease severity and adverse outcomes. These findings support its utility as a noninvasive serum biomarker for diagnosis, risk stratification and prognostic assessment in PAH-CHD patients.

## 1 Introduction

Pulmonary arterial hypertension associated with congenital heart disease (PAH-CHD) is a progressive, life-threatening disorder characterized by pulmonary vascular remodeling, perivascular infiltration of inflammatory cells, and, ultimately, right heart failure. Congenital heart disease (CHD) with a systemic-to-pulmonary shunt is among the most common causes of PAH in China (1). Epidemiological data reveal an annual incidence of 15.6 per million and an incidence of 2.2 new cases per million (2, 3). Secondary PAH occurs in 4%–28% of all patients with CHD and the incidence increases to approximately 30% among those with unrepaired CHD (4). Critically, the development of PAH accelerates clinical deterioration in patients with CHD, leading to increased heart failure-related hospitalizations and higher all-cause mortality.

PAH-CHD has a biphasic course: an early, potentially reversible phase in which timely shunt closure normalizes pulmonary hemodynamics and prevents irreversible remodeling and a late, irreversible phase in which shunt closure fails to lower pulmonary pressure and may trigger acute right ventricular failure or perioperative death (5). Thus, early detection and intervention are essential, but early-stage PAH-CHD is clinically silent: nonspecific symptoms such as dyspnea and fatigue delay diagnosis until irreversible hemodynamic deterioration occurs. Although right heart catheterization remains the diagnostic gold standard, its invasiveness, complication risk (6), and unsuitability for serial monitoring or population screening highlight the urgent need for noninvasive, quantifiable biomarkers to detect early pulmonary vascular pathology and monitor disease progression.

The a disintegrin-like and metalloproteinase with thrombospondin motifs (ADAMTS) enzymes are secreted zinc-dependent metalloproteases that critically regulate extracellular matrix homeostasis, angiogenesis, and inflammatory signaling in vascular tissues (7, 8). ADAMTS4, a well-characterized family member in cardiovascular contexts, is synthesized in smooth muscle cells of blood vessels and is secreted into the circulation where it remains measurable (9). ADAMTS4 regulates extracellular matrix degradation and turnover, particularly the degradation of versican. Robust evidence indicates that ADAMTS4 is a mediator of vascular instability: it is highly expressed in macrophage-rich areas of atherosclerotic plaques and unstable coronary plaques (10), and elevated plasma levels of ADAMTS4 correlate with plaque destabilization, acute coronary syndrome incidence, and increased disease severity (11). Similarly, ADAMTS4 expression is markedly upregulated in the dissected aortas and significantly elevated in the serum of patients with acute aortic dissection; thus, ADAMTS4 can serve as a diagnostic biomarker for aortic dissection (12, 13). These findings converge on a central pathobiological function: ADAMTS4 mediates degradation of the extracellular matrix during vascular remodeling, a process analogous to the pulmonary vascular lesions observed in PAH-CHD. However, no study has assessed serum ADAMTS4 levels in patients with PAH-CHD or its association with disease severity or prognosis.

Our prior work revealed time-dependent upregulation of lung ADAMTS4 in monocrotaline-induced PAH rats, paralleling increased pulmonary artery pressure and vascular remodeling (14, 15). In this study, we prospectively enrolled PAH-CHD patients, collected clinical data and peripheral blood, quantified serum ADAMTS4 concentrations, and linked these measurements to longitudinal follow-up outcomes to determine whether ADAMTS4 can serve as a blood biomarker for early diagnosis, severity assessment, and adverse prognosis prediction in PAH-CHD patients.

## 2 Materials and methods

### 2.1 Study design and ethical approval

This prospective cohort study was conducted from March 2020 to August 2025 at the First Affiliated Hospital of Gannan Medical University. The study protocol was reviewed and approved by the Institutional Ethics Committee of the hospital (Approval No.: LLSC-2022040601) and adhered strictly to the ethical principles outlined in the Declaration of Helsinki and the National Ethical Guidelines for Biomedical Research Involving Human Subjects in China. Written informed consent was obtained from all participants prior to enrollment.

### 2.2 Participant recruitment and group stratification

Consecutive adult patients who were diagnosed with PAH-CHD and those with CHD only and who did not receive pharmacological, interventional, or surgical treatment for pulmonary vascular disease were enrolled as case groups. The control group comprised age- and sex-matched healthy volunteers who were recruited from the hospital’s routine physical examination center. Healthy controls were required to meet two criteria: the absence of any history or clinical evidence of cardiovascular or pulmonary vascular disease and normal results across a standardized panel of laboratory tests, including blood count, hepatic and renal function indices, serum electrolytes, and fasting plasma glucose, obtained within the preceding 30 days.

The congenital heart defects included in the study included atrial septal defects, ventricular septal defects, and patent ductus arteriosus. PAH diagnosis adhered to the current hemodynamic definition: mean pulmonary arterial pressure (mPAP) ≥ 20 mmHg, pulmonary capillary wedge pressure (PCWP) ≤ 15 mmHg, and pulmonary vascular resistance (PVR) > 2 Wood units, all measured by right heart catheterization under resting conditions at sea level. For patients with Eisenmenger syndrome, a late-stage, irreversible form of PAH-CHD, a PAH diagnosis was made on the basis of ultrasound (bidirectional or right-to-left shunt, severe tricuspid regurgitation, right heart enlargement, and widened main pulmonary artery) and clinical manifestations (cyanosis, clubbing, and decreased exercise tolerance). The exclusion criteria included secondary pulmonary hypertension (e.g., due to chronic lung disease, left heart disease, or chronic thromboembolism), active pulmonary infection, moderate-to-severe hepatic impairment, an estimated glomerular filtration rate (eGFR) < 60 mL/min/1.73 m^2^ or active urinary tract infection, recent acute cardiovascular events, including acute coronary syndrome and acute aortic dissection, severe psychiatric illness, pregnancy or lactation, and cognitive or communication deficits precluding valid informed consent.

### 2.3 Clinical data collection

Demographic and clinical data, including sex, age, World Health Organization functional class (WHO-FC), and standardized laboratory parameters, were retrospectively extracted from the institutional electronic medical records system. Hematologic analyses were performed using the SYSMEX XE-2100 automated hematology analyzer and included white blood cell count (WBC), platelet count (PLT), plateletcrit (PCT), platelet distribution width (PDW), mean platelet volume (MPV), neutrophil-to-lymphocyte ratio (NLR), and absolute counts of neutrophils (NEUT), lymphocytes (LYM), and monocytes (MON). Biochemical assays were conducted on the Roche Cobas 8,000 platform and included triglyceride (TG), total cholesterol (TC), high-density lipoprotein cholesterol (HDL-C), low-density lipoprotein cholesterol (LDL-C), and serum uric acid (UA) levels.

All participants underwent standardized transthoracic echocardiography using the GE Vivid E95 color Doppler ultrasound system. The key quantitative parameters assessed included the left atrial end-diastolic diameter (LA-Dd), left atrial end-systolic diameter (LA-Ds), right atrial end-systolic diameter (RA-Ds), left ventricular end-diastolic diameter (LV-Dd), right ventricular end-diastolic diameter (RV-Dd), and main pulmonary artery diameter (MPA). In addition, the peak tricuspid regurgitant velocity (TVmax), stroke volume (SV), and left ventricular ejection fraction (LVEF) were measured to support the results of the hemodynamic assessment. Right heart catheterization was performed in patients with CHD both with and without clinically suspected PAH by interventional cardiologists in the cardiac catheterization laboratory. A Swan-Ganz catheter was used to obtain simultaneous measurements of mean right atrial pressure (mRAP), right ventricular systolic pressure (RVSP), and mean pulmonary arterial pressure (mPAP).

### 2.4 Blood sample collection and processing

After an overnight fast, 4.0 mL of venous blood was collected from the median cubital vein using a 22-gauge vacuum collection needle and transferred into sterile 5-mL serum separator tubes containing a clot activator. Tubes were gently inverted 8–10 times to ensure homogeneous mixing with the clot activator and allowed to stand undisturbed at room temperature (20–25 °C) for 30 min to permit complete coagulation. The samples were subsequently centrifuged at 1,500 × g for 20 min at 4 °C. The resulting supernatant serum was carefully aspirated under laminar flow conditions to minimize contamination, aliquoted into prelabeled, sterile 1.5-mL cryovials (200–300 μL per vial), and immediately flash-frozen on dry ice to prevent thermal degradation. Aliquots were stored at −80 °C within 30 min of serum separation to preserve analyte stability for subsequent biochemical analysis.

### 2.5 Serum biomarker quantification

Serum concentrations of ADAMTS4, N-terminal pro-B-type natriuretic peptide (NT-proBNP), and C-reactive protein (CRP) were quantified using commercially available sandwich enzyme-linked immunosorbent assay (ELISA) kits (Human ADAMTS4 ELISA Kit, Human NT-proBNP ELISA Kit, and Human CRP ELISA Kit; Cloud-Clone Corp., Wuhan, China) in strict accordance with the manufacturers’ instructions and internal quality control procedures. Briefly, 96-well microplates precoated with target-specific capture antibodies were incubated at 37 °C for 2 h, followed by blocking with assay buffer. Serum samples were diluted 1:5 in sample diluent, and serially diluted calibrator standards were prepared in parallel. Both samples and standards were added to the designated wells and incubated at 37 °C for 1 h. After three washes with phosphate-buffered saline containing 0.05% Tween-20 (PBST), biotin-conjugated detection antibodies were added and incubated for 1 h at 37 °C. Subsequently, streptavidin–horseradish peroxidase (HRP) conjugate was added, and the samples were incubated for 30 min at 37 °C. Color development was performed using a 3,3′,5,5′-tetramethylbenzidine (TMB) substrate solution, and the reaction was terminated with 2 mol/L sulfuric acid (H₂SO_4_). The absorbance was measured at 450 nm using a calibrated microplate reader. Standard curves were generated via four-parameter logistic (4PL) regression, and analyte concentrations in samples were interpolated from the respective standard curves.

### 2.6 Follow-up protocol and endpoint definition

PAH-CHD patients were followed prospectively every 6 months until August 1, 2025. Right heart catheterization confirmed PAH; its date served as the index date for follow-up. Follow-up included outpatient visits, inpatient records review, and telephone interviews. The primary endpoint was all-cause mortality, and the secondary endpoint was clinical deterioration, defined as the first hospitalization for acute decompensated right heart failure. The composite of death or hospitalization was used to define adverse outcomes for time-to-event analyses. Patients missing two consecutive follow-ups were considered lost to follow-up; survival time was censored at their last confirmed contact.

### 2.7 Statistical analysis

The statistical analyses were performed using R software (version 4.1.2). Prior to group comparisons, the normality of continuous variables was assessed using the Shapiro–Wilk test. To ensure robustness in determining the distribution characteristics, a conservative significance threshold of *p* = 0.01 was adopted. Continuous variables are reported as the mean ± standard deviation (SD) if normally distributed; otherwise, they are reported as the median and interquartile range (IQR). Categorical variables are expressed as frequencies and percentages (*n*, %). Group comparisons employed one-way ANOVA or the Kruskal–Wallis test for ≥3 groups, independent samples *t*-test or the Mann–Whitney *U* test for two groups, and the *χ*^2^ test or Fisher’s exact test for categorical outcomes. Spearman’s rank correlation was used to assess monotonic associations between serum ADAMTS4 levels and key clinical/biochemical variables. Univariate and multivariate logistic regression analyses were performed to identify independent risk factors associated with the presence of PAH in CHD patients. The diagnostic performance of serum ADAMTS4 for discriminating PAH-CHD was evaluated using receiver operating characteristic (ROC) curve analysis; the optimal cutoff value was determined by maximizing the Youden index, and the corresponding sensitivity and specificity were calculated. Patients were dichotomized accordingly (high vs. low ADAMTS4), and baseline characteristics and clinical outcomes were compared between groups. Survival analyses utilized the Kaplan–Meier method to estimate cumulative event rates, with between-group differences assessed by the log-rank test. Univariate and multivariate Cox proportional hazards regression analyses were performed to identify independent predictors of adverse clinical outcomes. The results are presented as hazard ratios (HRs) with corresponding 95% confidence intervals (CIs). All hypothesis tests were two-sided, and statistical significance was defined as *p* < 0.05.

## 3 Results

### 3.1 Clinical baseline characteristics of the study participants

Between March 2020 and August 2025, a total of 124 participants were enrolled: 46 with PAH-CHD, 47 with CHD, and 31 healthy controls (CON). Age and sex were matched across groups. Compared with the CON group, the PAH-CHD group had significantly higher NEUT, MONO, PDW, NLR, and UA levels and significantly lower PLT, PCT, TC, HDL-C, LDL-C, and TG levels (Table 1).

**Table 1:** Clinical baseline characteristics of the study subjects.

Variables	CON (*n* = 31)	CHD (*n* = 47)	PAH-CHD (*n* = 46)	*p-*value
Age (years)	49.00 (44.00, 56.00)	52.00 (36.00, 58.00)	56.00 (44.75, 64.50)	0.128
Gender				
Male	18.00 (58.06%)	17.00 (36.17%)	25.00 (54.35%)	0.097
Female	13.00 (41.94%)	30.00 (63.83%)	21.00 (45.65%)	
WHO-FC				
I/II	-	47.00 (100.00%)	18.00 (39.13%)	<0.001^*,#^
III/IV	-	0.00 (0.00%)	28.00 (60.87%)	
WBC (10^9^/L)	5.60 (4.89, 6.34)	5.86 (4.57, 7.10)	6.23 (4.93, 7.38)	0.324
PLT (10^9^/L)	247.00 (210.00, 283.50)	233.00 (187.00, 290.00)	206.00 (160.80, 249.00)	0.004^*,#^
NEUT (10^9^/L)	3.05 (2.44, 3.51)	3.23 (2.35, 4.59)	3.66 (2.86, 4.91)	0.037^*^
LYM (10^9^/L)	2.00 ± 0.52	2.55 ± 4.68	1.72 ± 0.77	0.101
MONO (10^9^/L)	0.39 (0.33, 0.48)	0.45 (0.40, 0.57)	0.49 (0.40, 0.63)	0.011^*^
PDW (%)	10.50 (9.55, 12.10)	11.25 (10.05, 15.93)	13.25 (11.00, 15.90)	0.004^*^
PCT (%)	0.25 ± 0.05	0.24 ± 0.05	0.20 ± 0.06	<0.001^*,#^
NLR	1.48 (1.12, 1.92)	1.77 (1.42, 2.54)	2.43 (1.47, 3.68)	0.008^*^
UA (μmol/L)	349.00 (295.50, 421.50)	313.00 (263.00, 374.00)	374.50 (325.00, 448.50)	0.002^*,#^
TC (mmol/L)	5.00 ± 0.68	4.17 ± 1.01	3.65 ± 0.83	<0.001^*,#^
HDL-C (mmol/L)	1.29 (1.04, 1.64)	1.20 (0.99, 1.35)	1.04 (0.91, 1.38)	0.050^*,#^
LDL-C (mmol/L)	3.04 ± 0.70	2.55 ± 0.85	2.26 ± 0.66	<0.001^*,#^
TG (mmol/L)	1.33 (0.95, 1.64)	1.18 (0.80, 1.91)	0.93 (0.73, 1.11)	0.005^*,#^

Statistical significance was defined as *p* < 0.05. Symbols denote group comparisons as follows: ^*^p indicates PAH-CHD versus CON; ^#^*p* indicates PAH-CHD versus CHD.

PAH-CHD patients were predominantly WHO-FC III/IV, whereas all CHD patients were WHO-FC I/II. Compared with the CHD group, the PAH-CHD group had significantly lower PLT, PCT, LDL-C, HDL-C, TC and TG levels and higher UA levels (Table 1). The hemodynamic parameters are presented in Supplementary Table 1. Compared with the CHD group, the PAH-CHD group had elevated mPAP and RVSP and markedly enlarged LA-Dd, LA-Ds, RA-Ds, RV-Dd, and MPA (*p* < 0.05), whereas the LV-Dd, SV and LVEF did not differ, which is consistent with right heart dominant remodeling.

### 3.2 Serum levels of ADAMTS4, NT-proBNP, and CRP in PAH-CHD patients

**Figure 1:**
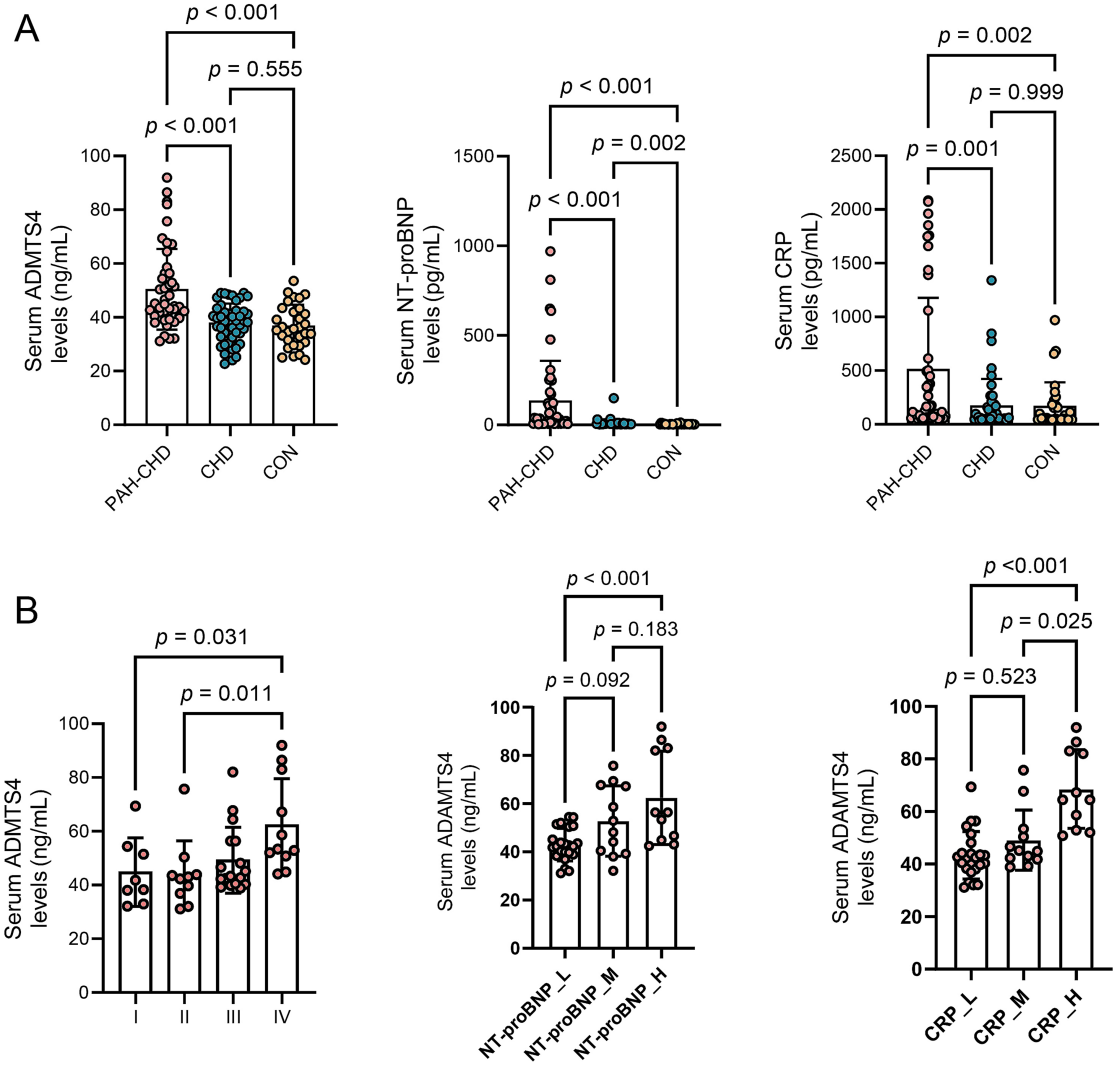
Serum levels of ADAMTS4, NT-proBNP, and CRP across study cohorts. **(A)** Comparison of serum ADAMTS4, NT-proBNP, and CRP concentrations among patients with PAH-CHD, patients with CHD, and healthy controls. **(B)** Stratification of serum ADAMTS4 levels in the PAH-CHD cohort according to WHO-FC class (I–IV) and NT-proBNP and CRP quartiles. CRP and NT-proBNP levels were stratified into three categories on the basis of quartile distribution: low (≤50th percentile), medium (51st–75th percentile), and high (>75th percentile).

ADAMTS4 levels were significantly higher in PAH-CHD patients than in both CHD patients and healthy controls (Figure 1A). NT-proBNP levels increased stepwise across the three groups, with the highest levels observed in the PAH-CHD group, intermediate levels in the CHD group, and the lowest levels in the healthy control group. Similarly, compared with those in both CHD patients and healthy controls, CRP levels were markedly elevated in PAH-CHD patients (Figure 1A). Given that WHO-FC, NT-proBNP, and CRP are well-established diagnostic and adverse prognostic indicators in PAH, PAH-CHD patients were further stratified into risk subgroups on the basis of WHO-FC class (I–IV), NT-proBNP, and CRP levels. NT-proBNP and CRP levels were categorized into three groups using quartile-based thresholds: low (≤50th percentile), medium (51st–75th percentile), and high (>75th percentile). As shown in Figure 1B, PAH-CHD patients with a higher WHO-FC class, higher NT-proBNP category, and higher CRP category had significantly elevated ADAMTS4 levels.

### 3.3 Correlation of serum ADAMTS4 levels with clinical parameters

**Figure 2:**
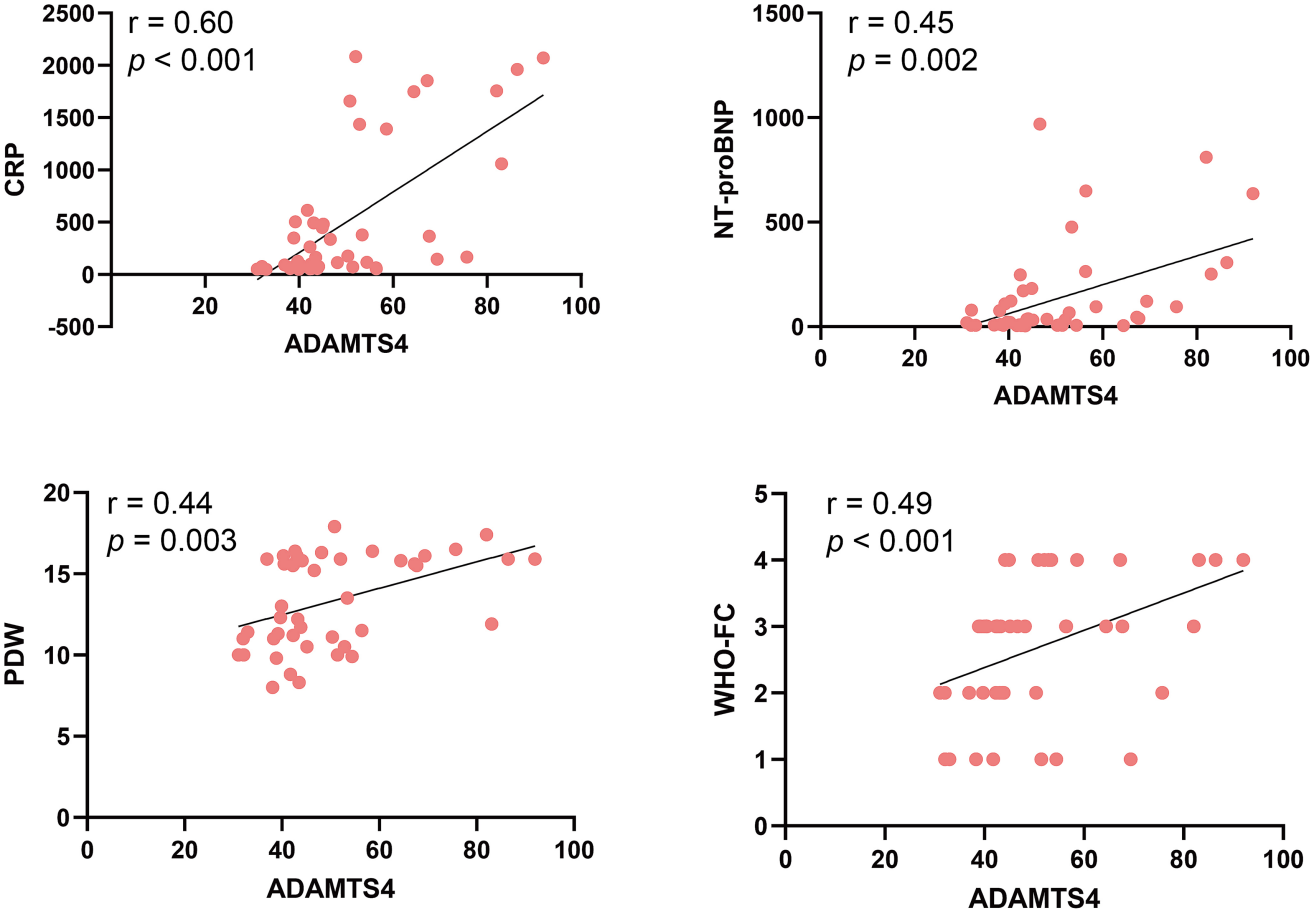
Correlation of serum ADAMTS4 with NT-proBNP, CRP, PDW and WHO-FC in patients with PAH-CHD. Correlations with clinical parameters were assessed using Spearman’s rank correlation.

Serum ADAMTS4 levels were significantly correlated with the following established markers of disease severity: moderate positive correlations with WHO-FC (*r* = 0.49, *p* < 0.001), NT-proBNP (*r* = 0.45, *p* = 0.002), and PDW (*r* = 0.44, *p* = 0.003) and a strong positive correlation with CRP (*r* = 0.60, *p* < 0.001; Figure 2). Prior evidence indicates that WHO-FC, NT-proBNP, CRP and PDW (16, 17) are associated with disease severity in PAH patients, thereby supporting serum ADAMTS4 as a biomarker of disease severity in PAH-CHD patients.

### 3.4 Diagnostic performance of ADAMTS4

**Figure 3:**
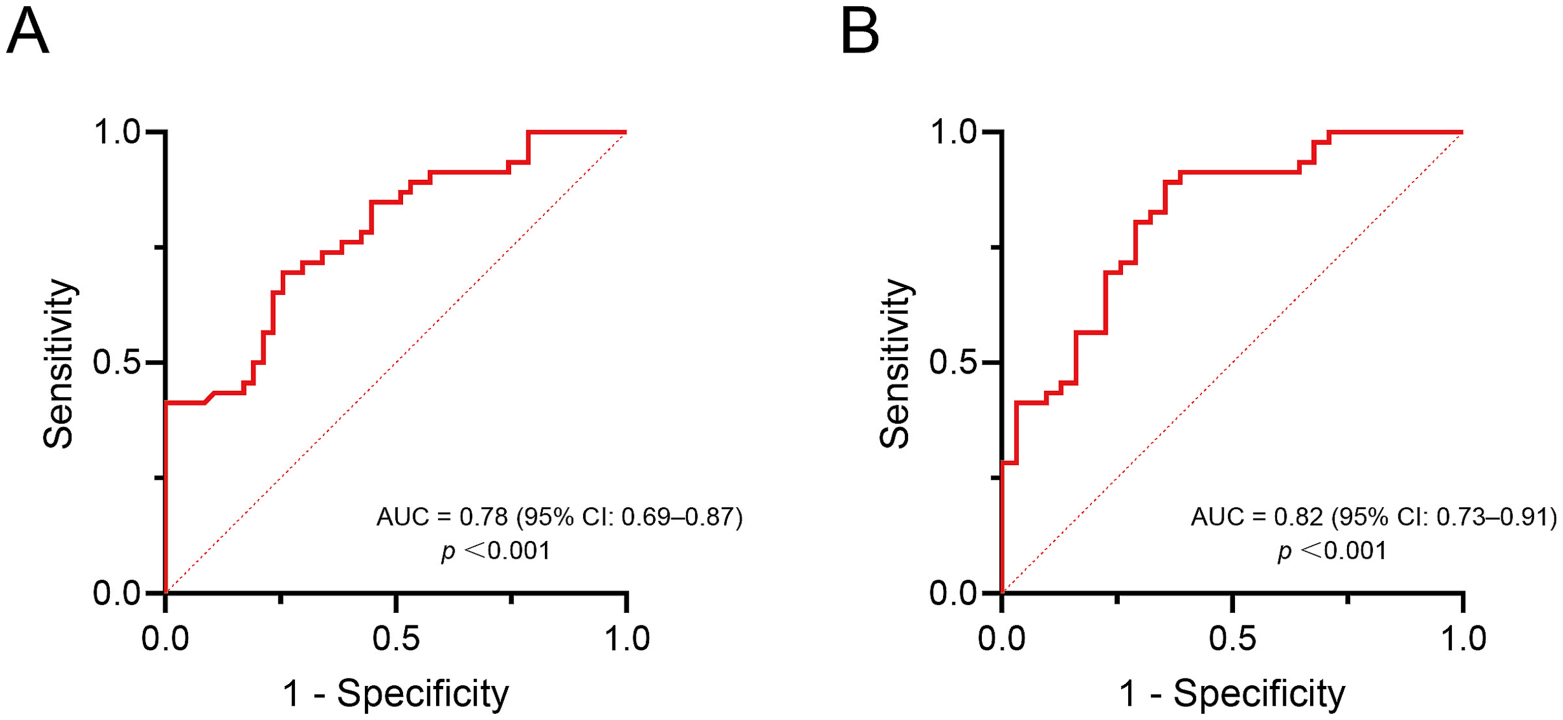
Diagnostic performance of serum ADAMTS4 for differentiating PAH-CHD from CHD **(A)** and from CON **(B)**. The ROC curves are shown, with corresponding AUC values and 95% confidence intervals.

ROC curve analysis was used to evaluate the diagnostic utility of serum ADAMTS4 for differentiating patients with PAH-CHD from those with CHD alone. ADAMTS4 achieved an area under the curve (AUC) of 0.78 (95% CI: 0.69–0.87), indicating moderate discriminative accuracy. At the optimal cutoff of 42.20 ng/mL, the sensitivity was 70.2%, the specificity was 74.5%, and the overall accuracy was 72.3% (Supplementary Table 2, Figure 3A). A cutoff value of 42.20 ng/mL was applied in subsequent analyses. When PAH-CHD patients were distinguished from healthy controls, the diagnostic performance of ADAMTS4 improved (AUC = 0.82; 95% CI: 0.73–0.91). At the optimized cutoff of 37.78 ng/mL, the sensitivity increased to 89.4%, the specificity was 64.5%, and the accuracy reached 79.5% (Supplementary Table 2, Figure 3B).

### 3.5 Clinical characteristics across ADAMTS4 subgroups

Stratifying PAH-CHD patients at the 42.2 ng/mL cutoff generated ADAMTS4-high (ADAMTS4 ≥ 42.2 ng/mL, *n* = 32) and ADAMTS4-low (ADAMTS4 < 42.2 ng/mL, *n* = 14) groups. The ADAMTS4-high group had higher CRP, NLR, WHO-FC III/IV, NT-proBNP, and PDW levels (*p* < 0.05) and lower LYM and HDL-C levels (Table 2). Consequently, these findings suggest that increased ADAMTS4 levels are associated with an elevated inflammatory response and impaired cardiac function.

**Table 2:** Clinical characteristics of patients stratified by ADAMTS4 levels.

Variables	ADAMTS4-high (*n* = 32)	ADAMTS4-low (*n* = 14)	*p-*value
Age (years)	57.00 (48.50, 66.00)	53.50 (45.00, 59.25)	0.232
Gender			
Male	19.00 (59.38%)	6.00 (42.86%)	0.349
Female	13.00 (40.62%)	8.00 (57.14%)	
WHO-FC			
I/II	9.00 (28.13%)	9.00 (64.29%)	0.016
III/IV	23.00 (71.87%)	5.00 (35.71%)	
NT-proBNP (pg/mL)	183.20 ± 254.7	36.73 ± 42.57	<0.001
CRP (ng/mL)	299.30 (93.23, 1,401.74)	76.10 (57.47, 116.48)	0.010
WBC (10^9^/L)	6.23 (4.98, 7.27)	6.16 (4.83, 7.71)	0.943
PLT (10^9^/L)	197.81 ± 65.54	215.14 ± 47.38	0.378
NEUT (10^9^/L)	3.71 (3.40, 5.06)	3.27 (2.44, 4.49)	0.257
LYM (10^9^/L)	1.52 ± 0.69	2.15 ± 0.78	0.009
MONO (10^9^/L)	0.54 ± 0.21	0.50 ± 0.13	0.476
NLR	2.77 (1.65, 4.83)	1.50 (1.37, 2.42)	0.009
PDW (%)	15.55 (11.55, 16.05)	11.15 (10.00, 12.82)	0.014
PCT (%)	0.20 ± 0.07	0.20 ± 0.05	0.756
UA (μmol/L)	377.00 (337.75, 461.50)	363.50 (334.25, 405.75)	0.424
TC (mmol/L)	3.54 ± 0.75	3.91 ± 0.98	0.182
HDL-C (mmol/L)	1.04 ± 0.37	1.32 ± 0.33	0.025
LDL-C (mmol/L)	2.22 ± 0.64	2.35 ± 0.74	0.561
TG (mmol/L)	0.93 (0.74, 1.09)	0.93 (0.80, 1.17)	0.918

### 3.6 Risk factors associated with the presence of PAH in CHD patients

Univariate logistic regression analyses revealed that PLT, PCT, TC, and TG were significantly associated with a lower likelihood of PAH-CHD. In contrast, higher levels of ADAMTS4 (OR = 1.138; 95% CI: 1.065–1.216; *p* < 0.001), NT-proBNP, CRP, and UA were significantly associated with an increased likelihood of PAH-CHD (Table 3). After multivariable adjustment for clinical and biochemical confounders, including NT-proBNP, CRP, PLT, PCT, TC, TG, and UA, elevated ADAMTS4 remained an independent factor associated with the presence of PAH-CHD (OR = 1.189; 95% CI: 1.036–1.365; *p* = 0.014).

**Table 3:** Logistic regression analysis for identifying risk factors for PAH-CHD.

Variables	Univariate analysis			Multivariate analysis		
	OR	95% CI	*p*-value	OR	95% CI	*p*-value
ADAMTS4	1.138	1.065–1.216	<0.001	1.189	1.036–1.365	0.014
NT-proBNP	1.038	1.011–1.067	0.006	1.088	1.009–1.172	0.027
CRP	1.002	1.001–1.003	0.009	0.999	0.995–1.002	0.437
PLT	0.991	0.987–0.997	0.031	1.014	0.980–1.049	0.431
PCT	0.001	0.000–0.007	0.005	0.001	0–2.252	0.489
UA	1.009	1.004–1.014	0.001	1.019	1.004–1.033	0.010
TC	0.597	0.375–0.950	0.030	0.863	0.369–2.022	0.735
TG	0.243	0.093–0.631	0.004	0.260	0.058–1.174	0.080

### 3.7 Prognostic value of serum ADAMTS4 in PAH-CHD

**Figure 4:**
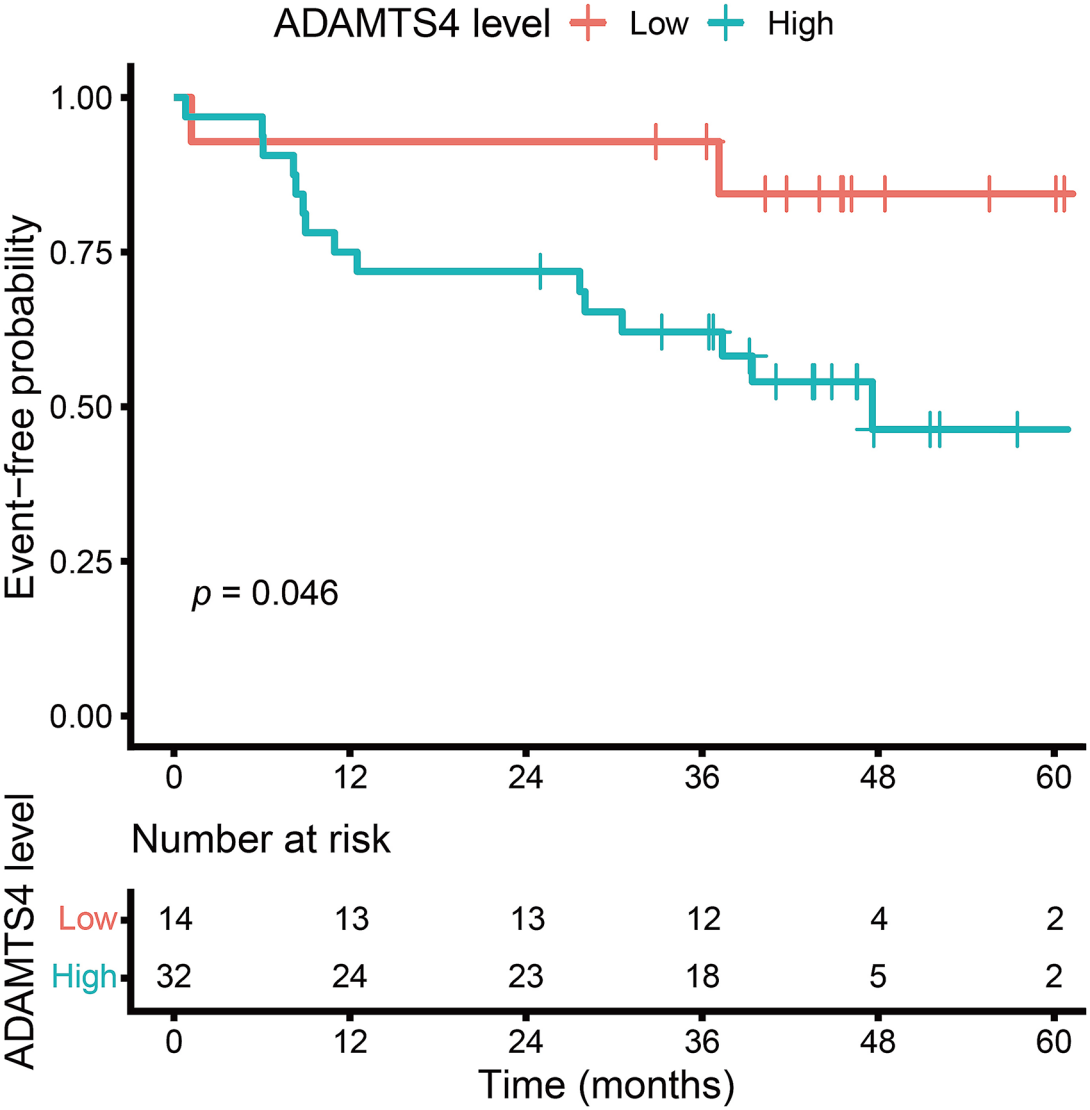
Kaplan–Meier survival curves comparing survival across the ADAMTS4 subgroup: ADAMTS4-high group, ADAMTS4 level ≥42.2 ng/mL; ADAMTS4-low group, ADAMTS4 level < 42.2 ng/mL.

Over a median follow-up of 40.5 (interquartile range, 27.4–47.5) months, 19 of the 46 PAH-CHD patients (41.3%) reached the composite adverse endpoints—eight deaths and 11 hospitalizations due to worsening right heart failure. Kaplan–Meier survival analysis revealed significantly shorter event-free survival in the ADAMTS4-high group than in the ADAMTS4-low group (log-rank test, *p* = 0.046; Figure 4). Univariate Cox proportional hazards regression revealed that ADAMTS4 was a significant predictor of adverse clinical outcomes (HR = 1.049; 95% CI: 1.022–1.077; *p* < 0.001). Importantly, this association persisted after multivariable adjustment for established clinical and noninvasive prognostic variables, including WHO-FC, NT-proBNP, CRP, HDL-C, and PDW (HR = 1.038; 95% CI: 1.007–1.071; *p* = 0.017; Table 4).

**Table 4:** Multivariate Cox proportional hazards regression analysis of prognostic factors in patients with PAH-CHD.

Variables	HR	95% CI	*p-*value
ADAMTS4 (ng/mL)	1.038	1.007–1.071	0.017
WHO-FC III/IV	0.295	0.061–1.415	0.127
NT-proBNP (pg/mL)	1.000	0.997–1.002	0.854
CRP (ng/mL)	1.000	0.999–1.001	0.910
PDW (%)	1.028	0.834–1.266	0.797
HDL-C (mmol/L)	1.307	0.301–5.682	0.721

## 4 Discussion

This study demonstrated that serum ADAMTS4 levels are significantly elevated in patients with PAH-CHD and are associated with clinical markers of disease severity. ADAMTS4 shows good discriminatory ability for distinguishing PAH-CHD from CHD and is an independent predictor of adverse clinical outcomes, including all-cause mortality and heart failure-related hospitalization, supporting its utility as a biomarker for early diagnosis, disease severity assessment, and adverse prognosis prediction in patients with PAH-CHD.

PAH is a progressive cardiopulmonary disorder characterized by pathological remodeling of distal pulmonary arterioles, chronic perivascular inflammation, and eventual right ventricular failure. Distal pulmonary arteriolar remodeling constitutes the central histopathological hallmark, driven by dysregulated proliferation and migration of pulmonary artery smooth muscle cells, immune cell infiltration, and perturbed turnover of the extracellular matrix, including excessive degradation and aberrant deposition (18). Our previous study demonstrated that an inflammatory/immune response was detectable at an early stage of PAH development and that dysregulated chemokine signaling was involved in the onset and progression of PAH (19). Consistently, Bolayır et al. reported that circulating inflammatory markers, CRP and IL-34, were elevated in PAH patients and moreover, were associated with PAH severity and right ventricular dysfunction, highlighting the potential of IL-34 as a diagnostic and prognostic biomarker (20). ADAMTS4 is a secreted, zinc-dependent metalloproteinase with well-established substrate specificity for key extracellular matrix components, such as aggrecan and versican (9). Its transcription is tightly regulated by multiple inflammatory mediators, including IL-1, TGF-β, TNF-α, and IL-6, mainly via NF-κB and SMAD-dependent pathways (21, 22). Importantly, these cytokines are consistently overexpressed in lung tissue from PAH animal models and elevated in serum from PAH patients (14, 15, 23). ADAMTS4 is likely not only a downstream epiphenomenon of inflammation but also an active, mechanistically engaged node within the inflammation–extracellular matrix–vascular remodeling axis.

In support of this functional linkage, prior evidence has revealed that activated monocytes/macrophages are major cellular sources of ADAMTS4 in atherosclerotic plaques and plasma (10, 24). Additionally, ADAMTS4 overexpression is localized to vascular smooth muscle cells in human aortic aneurysm and chronic dissection tissues (13). Histopathological studies have reported perivascular infiltration by macrophages, mast cells, and T lymphocytes in remodeled pulmonary vessels and plexiform lesions of PAH patients (23, 25). Consistent with these findings, our data revealed significant positive correlations between serum ADAMTS4 levels and the systemic inflammatory marker CRP, suggesting that inflammatory cues in the pulmonary vasculature may drive ADAMTS4 expression and secretion from pulmonary vascular smooth muscle cells and infiltrating macrophages, thereby fuelling extracellular matrix dysregulation and maladaptive vascular remodeling.

The secretion and activity of ADAMTS4 depend on C-mannosylation (26). Reflecting its pathophysiological relevance, circulating ADAMTS4 has emerged as a severity-associated biomarker across multiple cardiovascular conditions: it is markedly elevated in acute coronary syndrome and predicts plaque destabilization (24, 27). Likewise, in acute aortic dissection, ADAMTS4 levels correlate strongly with the diagnosis and risk of aneurysmal progression or rupture (12, 28). In addition, ADAMTS4 is upregulated in the injured heart and has been identified as a new biomarker for detecting cardiac injury in adults (29). In the present study, serum ADAMTS4 levels were elevated in patients with PAH-CHD and were positively correlated with established clinical indicators of disease severity, including NT-proBNP, CRP, WHO-FC and PDW. Notably, compared with the ADAMTS4-low group, the ADAMTS4-high group (≥42.2 ng/mL) had higher WHO-FC III/IV, NT-proBNP, CRP, NLR, and PDW and lower LYM and HDL-C levels, further reinforcing its clinical validity as a quantitative marker of disease severity. Collectively, these findings substantiate ADAMTS4 as a clinically meaningful biomarker of disease severity in PAH-CHD patients.

Diagnostic and prognostic performance was rigorously evaluated. ROC analysis revealed that ADAMTS4 distinguished PAH-CHD patients from healthy controls, with an AUC of 0.82, and differentiated PAH-CHD patients from CHD patients, with an AUC of 0.78. Multivariate logistic regression revealed that elevated ADAMTS4 is an independent risk factors associated with the presence of PAH in CHD patients. Kaplan–Meier survival analysis revealed significantly shorter event-free survival in the ADAMTS4-high group than in the ADAMTS4-low group. Importantly, Cox proportional hazards modeling confirmed that ADAMTS4 is an independent predictor of adverse clinical outcomes after multivariable adjustment for established clinical and noninvasive prognostic variables, including WHO-FC, NT-proBNP, CRP, HDL-C, and PDW, highlighting that its predictive power was not dependent on traditional prognostic indicators.

Several limitations merit consideration. First, this was a single-center observational cohort study with a relatively small sample size. The limited sample size may have constrained statistical power, reducing the precision of effect estimates and preventing meaningful subgroup or sensitivity analyses. Additionally, the single-center design may introduce selection bias and limit the external generalizability of our findings. Second, this study is cross-sectional in nature. Therefore, logistic regression analyses can only identify factors associated with the presence of PAH in CHD patients, rather than predict future disease onset. Third, the median follow-up duration was relatively short and the number of composite events was limited. This analysis should be considered exploratory, and the observed associations between serum ADAMTS4 concentration and long-term clinical endpoints remain to be confirmed in prospective, multicenter, longitudinal investigations.

## 5 Conclusion

In summary, this study provides the first evidence that ADAMTS4 is significantly elevated in the peripheral circulation of patients with PAH-CHD and is associated with both disease severity and adverse clinical outcomes. These findings support the potential utility of serum ADAMTS4 as a novel, noninvasive biomarker for diagnosis, risk stratification, and prognostic assessment in patients with PAH-CHD.

## Data Availability

The original contributions presented in the study are included in the article/Supplementary material, further inquiries can be directed to the corresponding author/s.
