# Supplementary material for: ADAMTS4 is a serum biomarker for pulmonary arterial hypertension associated with congenital heart disease
Source: Front Med (Lausanne). 2026 Jul 22;13:1863052. doi: 10.3389/fmed.2026.1863052 (PMC13438119; doi:10.3389/fmed.2026.1863052)
Supplement: Supplementary file 1 [file Table_1.DOCX]

Supplementary Material

# Supplementary Tables

Supplementary Table 1. Cardiac hemodynamic parameters

| Variables | PAH-CHD (n = 46) | CHD (n = 47) | *P* value |
| --- | --- | --- | --- |
| Echocardiography | | | |
| LA-Dd (mm) | 40.00 (33.00, 46.50) | 33.00 (30.00, 37.00) | <0.001 |
| LA-Ds (mm) | 43.50 (37.00, 49.25) | 37.00 (35.00, 41.00) | <0.001 |
| LV-Dd (mm) | 42.50 (40.00, 51.25) | 45.00 (40.00, 48.00) | 0.767 |
| RA-Ds (mm) | 48.00 (39.00, 54.00) | 36.00 (34.00, 42.00) | <0.001 |
| RV-Dd (mm) | 29.00 (23.75, 39.25) | 20.00 (20.00, 26.00) | <0.001 |
| MPA (mm) | 36.00 (30.00, 42.00) | 24.00 (23.00, 28.00) | <0.001 |
| TVmax (m/s) | 3.85 ±0.77 | 2.59 ±0.48 | <0.001 |
| LVEF (%) | 60.00 (54.75, 66.25) | 64.00 (60.00, 67.00) | 0.064 |
| SV (ml/b) | 56.10 ±22.62 | 62.76 ±21.07 | 0.063 |
| Right heart catheterization | | | |
| mPAP (mmHg) | 32.03 ±12.34 | 14.94 ±3.51 | <0.001 |
| RVSP (mmHg) | 54.00 (44.00, 83.75) | 32.00 (27.00, 37.00) | <0.001 |
| mRAP (mmHg) | 6.85 ±5.25 | 4.58 ±2.52 | 0.086 |

Supplementary Table 2. Diagnostic performance of ADAMTS4

| Variables | AUC | 95% CI | *P* value | Cutoff value | Sensitivity | Specificity | AC | Comparison |
| --- | --- | --- | --- | --- | --- | --- | --- | --- |
| ADAMTS4 | 0.78 | 0.69-0.87 | <0.001 | 42.20 ng/mL | 70.21% | 74.47% | 72.34% | PAH-CHD vs CHD |
| ADAMTS4 | 0.82 | 0.73-0.91 | <0.001 | 37.78 ng/mL | 89.36% | 64.52% | 79.49% | PAH-CHD vs CON |
